# A complete DNA sequence map of the ovine Major Histocompatibility Complex

**DOI:** 10.1186/1471-2164-11-466

**Published:** 2010-08-10

**Authors:** Jianfeng Gao, Ka Liu, Haibo Liu, Hugh T Blair, Gang Li, Chuangfu Chen, Pingping Tan, Runlin Z Ma

**Affiliations:** 1 School of Life Sciences, Shihezi University, Xinjiang 832007, China; 2 Institute of Genetics and Developmental Biology, Chinese Academy of Science, Beijing 100101, China; 3 Institute of Veterinary Animal and Biomedical Sciences, Massey University, New Zealand; 4 Graduate School of Chinese Academy of Sciences, Beijing 100149, China

## Abstract

**Background:**

The ovine Major Histocompatibility Complex (MHC) harbors clusters of genes involved in overall resistance/susceptibility of an animal to infectious pathogens. However, only a limited number of ovine MHC genes have been identified and no adequate sequence information is available, as compared to those of swine and bovine. We previously constructed a BAC clone-based physical map that covers entire class I, class II and class III region of ovine MHC. Here we describe the assembling of a complete DNA sequence map for the ovine MHC by shotgun sequencing of 26 overlapping BAC clones.

**Results:**

DNA shotgun sequencing generated approximately 8-fold genome equivalent data that were successfully assembled into a finished sequence map of the ovine MHC. The sequence map spans approximately 2,434,000 nucleotides in length, covering almost all of the MHC loci currently known in the sheep and cattle. Gene annotation resulted in the identification of 177 protein-coding genes/ORFs, among which 145 were not previously reported in the sheep, and 10 were ovine species specific, absent in cattle or other mammals. A comparative sequence analyses among human, sheep and cattle revealed a high conservation in the MHC structure and loci order except for the class II, which were divided into IIa and IIb subregions in the sheep and cattle, separated by a large piece of non-MHC autosome of approximately 18.5 Mb. In addition, a total of 18 non-protein-coding microRNAs were predicted in the ovine MHC region for the first time.

**Conclusion:**

An ovine MHC DNA sequence map was successfully assembled by shotgun sequencing of 26 overlapping BAC clone. This makes the sheep the second ruminant species for which the complete MHC sequence information is available for evolution and functional studies, following that of the bovine. The results of the comparative analysis support a hypothesis that an inversion of the ancestral chromosome containing the MHC has shaped the MHC structures of ruminants, as we currently observed in the sheep and cattle. Identification of relative large numbers of microRNAs in the ovine MHC region helps to provide evidence that microRNAs are actively involved in the regulation of MHC gene expression and function.

## Background

The sheep is one of the major domestic animal species for human meat protein, milk, and its wool is a source of industrial fiber. The Major Histocompatibility Complex (MHC) of the sheep, also designated as ovine Lymphocyte Antigen (OLA), harbors clusters of immunological genes involved in overall resistance/susceptibility of the animal to infectious diseases [1-3]. A number of agriculturally important traits, especially those related to disease resistance to various pathogenic viruses, bacteria and parasites, are closely linked to genes in the MHC [4-6]. Furthermore, genetic loci in the MHC are organized to form distinct functional clusters as class I, class II, and class III, which show a considerable level of conservation among mammal species [7-19]. The importance of sheep MHC molecules in disease resistance [6,20-23] and the associated structure features in artiodactyls have led to increased studies on the sheep MHC [5,21,24-26]. However, the detailed sequence information for ovine MHC is not sufficiently adequate, and only a small number of ovine MHC genes have been identified as compared to those in sheep and cattle.

Studies of the ovine MHC also help to provide valuable information on comparative genome evolution in mammals. The extreme high level of polymorphism observed for MHC loci may be a result of the evolutionary consequences of intensive interactions between infectious pathogens and the host defensive system [7]. Haplotype difference among different breeds adds another level of complexity. Previous studies on the OLA have largely been focused on the gene content and polymorphisms of the class region [27-32]. Based on the genetic linkage studies, the ovine MHC seems to have a special feature in that the class II has been divided into two sub-regions, similar to that of bovine [33-37]. However, with the limited sequence information available for the sheep, such structural features can not be adequately assessed by comparison with that of the cattle.

We previously constructed a BAC-clone-based physical map of the ovine MHC for Chinese merino fine-wood sheep [26], a valued sheep breed predominant in Northwest China especially in the Xinjiang Uygur autonomous region. The DNA used for BAC library construction was obtained from a heterozygous Chinese merino male, this animal being a merino ram that shares less than 1/32 of the blood from a local Chinese sheep breed. The BAC clone source we established facilitates the physical map construction for sheep MHC and for whole sheep genome, which serve as a reference frame work for subsequent sequencing. To facilitate the DNA sequencing, a BAC clone gap which previously existed between locus *Notch4 *and *Btnl2 *was successfully closed by addition of two more overlapping BAC clones [38].

Here we describe our work on sequencing of the entire ovine MHC by shotgun sequencing of the 26 BAC clones, assembling of the sequence data into a finished DNA sequence map as guided by the physical map, and the sequence analysis that resulted in identification and annotation of 177 genes and 18 microRNAs in ovine MHC region.

## Results and Discussion

DNA shotgun sequencing was successfully performed for 26 overlapping BAC clones, generating approximately 8-fold coverage of the genome equivalent data. The fully-assembled sequences for all of the BAC clones were deposited into GenBank with accession numbers FJ986852 - FJ985877 (Table 1). The quality of the sequence determined was adequate, with an estimated error rate less than 0.025% for most of the BAC clones. An average of 1.3 gaps existed per BAC clone, mostly due to highly repetitive sequence. A gap here refers to a stretch of DNA for which the exact nucleotide base identity (A, G, T, or C) remain ambiguous after resequencing, represented by a tandem number of "N" between the determined sequences.

**Table 1:** Assembly of 26 BAC-clone based DNA sequences covering entire Ovine MHC region

BAC clone ID	GenBank Accession Number	Insert length (bp)	**Average coverage**^**a**^	**Single-base error probability**^**b**^	**Reads number**^**c**^	High repeat sequence	**Scaffolds**^**d**^	**No. of Gaps inside**^**e**^	No. of Gaps outside
271H22	FJ985865	159959	7.93491	1.118 × 10^-4^	2974	No	1	0	0
304C7	FJ985867	134586	8.0733	2.127 × 10^-4^	2509	Yes	1	1	0
142M19	FJ985860	134479	8.11391	4.257 × 10^-4^	2715	Yes	1	2	0
373D13	FJ985872	172485	8.15691	5.308 × 10^-4^	3311	No	1	3	0
283N15	FJ985866	155021	7.165	0.268 × 10^-4^	2472	No	1	0	0
222G18	FJ985862	167309	7.78757	0.790 × 10^-4^	2783	No	1	0	0
55L9	FJ985854	145292	8.22565	0.195 × 10^-4^	2941	No	1	0	0
197N2	FJ985876	90102	6.50404	0.488 × 10^-4^	1438	No	1	2	0
429P24	FJ985873	198404	9.02502	3.497 × 10^-4^	4009	No	1	2	0
225J15	FJ985863	139059	7.9057	68.30 × 10^-4^	2335	Yes	1	1	0
453O11	FJ985874	143310	8.05201	0.802 × 10^-4^	2473	No	1	2	0
63M17	FJ985856	129209	8.52801	37.780 × 10^-4^	2394	No	1	1	0
163P3	FJ985861	165447	7.55517	1.049 × 10^-4^	2833	No	1	0	0
119K1	FJ985858	156603	7.75008	0.309 × 10^-4^	2665	No	1	2	0
349I12	FJ985871	149708	8.4984	7.600 × 10^-4^	2994	No	1	2	0
345B17	FJ985869	134643	7.69046	10.58 × 10^-4^	2736	No	2	0	1
68G10	FJ985857	165531	8.14164	6.535 × 10^-4^	3681	Yes	1	1	0
346G21	FJ985870	138311	8.67944	9.059 × 10^-4^	2807	No	1	1	0
44I10	FJ985853	134434	8.32584	16.47 × 10^-4^	2705	No	1	2	0
282P19	FJ985875	174317	7.27217	0.490 × 10^-4^	2989	No	2	1	1
239C1	FJ985864	142287	7.9438	0.736 × 10^-4^	2940	No	1	2	0
141C4	FJ985859	160633	7.65743	0.624 × 10^-4^	2992	No	1	1	0
374N21	FJ985877	83460	9.112	3.860 × 10^-4^	3648	No	2	1	1
21H3	FJ985852	119055	8.13723	0.380 × 10^-4^	2157	No	1	1	0
304D17	FJ985868	140735	8.14013	4.291 × 10^-4^	2599	No	1	1	0
58G13	FJ985855	135958	8.827674	2.187 × 10^-4^	3845	No	2	4	0

^a ^Defined as a ratio between total number of base pairs sequenced and total number of base pairs of the inserts in a given BAC clone.

^b ^Error probability of a particular base call, corresponding to a quality value as determined by the equation:

Q = -10log_10_(P_e_), where P_e _is the error probability.

^c ^The total number of shotgun DNA sequencing reactions performed for a given BAC clone.

^d ^In genomic mapping, a series of contigs that are in the right order but not necessarily connected in one continuous stretch of sequence.

^e ^The number of regions where the exact nucleotide base (G, A, T, or C) could not determined, represented by a strips of "N" in a given BAC clone.

**Figure 1:**
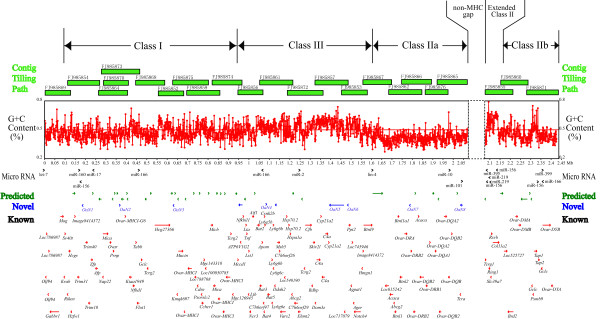
**A feature map of Ovine MHC sequence**. The map spans 2,434,000 nucleotide bases in length, containing 177 protein-coding genes/ORFs and 18 miRNA coding genes. Each locus is represented by an arrow or arrow head, and annotated according to type, orientation, and location within the MHC. The tiling path of the sequenced BACs and the MHC structure are shown on the top. **Micro RNA **(18 shown): Identified gene that has high sequence homology with conserved gene encoding the functional mircoRNA in other species, noted following the given name of that species. **Predicted **(36 shown): Gene that either has high sequence similarity to that of the predicted gene in other species, or has a predicted ORF but no high sequence homologies with ESTs of Ovine or other species. **Novel **(10 shown): Ovine-specific gene identified with a defined open reading frame (ORF) that has not been found in any other species to date. The novel genes are annotated with *OaN1 *to *OaN8 *(*Oa *for *Ovis aries*; N for novel; another two with no cloning data, no annotation), from left to the right of the map. **Known **(131 in total): The functional genes previously annotated in Bovine, Ovine or other species.

A complete DNA sequence map of the ovine MHC was successfully assembled as guided by the BAC clone physical map (Figure 1). The map spans approximately 2,434,000 nucleotide bases in length, covering almost all MHC loci currently known for both ovine and bovine species. The finished sequence map was discontinuous, as expected from the physical map. The major sequence segment spans approximately 2,071,000 nucleotide bases, harboring class I, class III, and class IIa of the ovine MHC. The shorter sequence segment spans approximately 363,000 nucleotide bases, harboring loci in the class IIb region and extending into the non-MHC region.

Sequence analysis resulted in the identification and annotation of 177 protein-coding genes/ORFs in the ovine MHC (Figure 1, Additional table 1). Of the 177 ovine genes identified, 131 were homologous to previously annotated genes in cattle, sheep or other mammal species, 36 matched to the predicted but not yet annotated genes in the cattle, and 10 were ovine species specific, having not been found in human, mouse, cattle or other mammal sequences. The location, transcriptional orientation, and relative size of the identified genes were determined (Figure 1). Among the genes identified, a total of 145 identified ovine genes were reported for the first time by this study. The ovine-specific genes were temporally nominated as *"OaN*" followed by a numeric number, where "Oa" is abbreviation for *Ovis aries*, and "N" for novel (Additional file1). Preliminary experiments confirmed the mRNA transcripts for 4 of the predicted ovine-specific genes (data not shown). The distribution of these novel genes seems to be random throughout the ovine MHC region. It is interesting to notice that a multiple DQ loci (DQ cluster) were identified, each with different orientation of transcription, when compared with those of other sheep breeds [39,40]. Such difference may be due either to breed or haplotype differences, as a subsequence of differential gene duplication [41].

An additional 18 genes encoding micro RNAs were identified by software prediction in an effort to search for non-protein-coding genes/components using the Rfam database analysis tools (Table 2). The orientation and distribution of these micro RNAs showed a randomized pattern throughout MHC region. This is the first time that a relatively large number of microRNAs have been identified in ovine MHC region. Given the functional importance of microRNAs for regulating gene expression by mRNA cleavage or repression, this preliminary finding help to provide evidence that microRNAs may be actively involved in the MHC response to pathogens in general.

**Table 2:** List of non-protein-codning microRNA genes identified in MHC by Rfam analysis

Gene Name	Rfam Accession No.	Start coordinate	End coordinate	Orientation	**Score**^**a**^
let-7	RF00027	35	51	+	34.2
miR-160	RF00247	173327	173343	+	34.2
miR-156	RF00073	180706	180722	-	34.2
miR-17	RF00051	243137	243153	-	34.2
miR-166	RF00075	465267	465295	+	34.2
miR-166	RF00075	1062114	1062131	-	36.2
miR-2	RF00047	1243062	1243078	+	34.2
lin-4	RF00052	1608564	1608580	+	34.2
miR-10	RF00104	1978042	1978059	+	36.2
miR-101	RF00253	2046800	2046816	+	34.2
miR-395	RF00451	2082358	2082373	-	32.2
miR-156	RF00073	2084006	2084023	-	36.2
miR-219	RF00251	2098884	2098955	-	127
miR-219	RF00251	2098930	2098950	-	42.1
miR-156	RF00073	2131290	2131307	-	36.2
miR-156	RF00073	2339014	2339030	+	34.2
miR-166	RF00075	2348202	2348217	-	32.2
miR-399	RF00445	2354071	2354086	+	32.2

^a ^The scores are bits (logs-odds) scores which represent the log of the probability of the query given the model over the probility of random sequence given the model.

**Figure 2:**
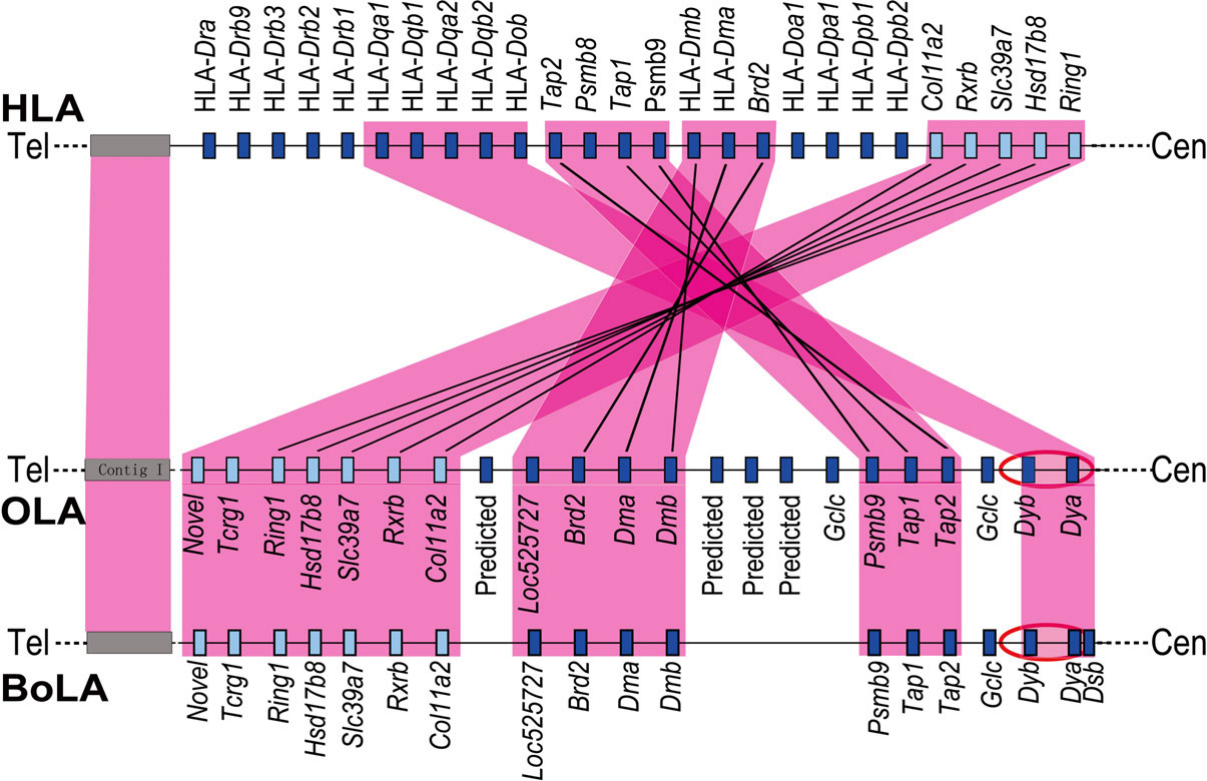
**Gene order comparisons for the selected class II loci from HLA, OLA, and BoLA**. Genetic loci in class II region were compared by aligning HLA, OLA and BoLA at telemere→centromere orientation. The orthologous loci were linked by solid lines. Solid and open box represent the selected class II loci and non-MHC loci, respectively. Shaded carmine boxes indicate regions of conservation among species. Red ellipses indicate the potential breaking points.**Tel**: Telomere, **Cen**: Centromere.

Sequence alignments among the human, sheep, and cattle MHC showed an overall conservation, with the level of homology reaching over 85% for the MHC class I, class III, and part of class II regions. The major difference in the MHC structures was found in the class II region. In human it was a continuous segment with no interruption, while in the sheep and cattle it was divided into IIa and IIb subregions by a large piece of non-MHC autonomic insertion. In addition, the gene order of class IIb in both ovine and bovine regions showed an opposite orientation relative to that of human (Figure 2).

Analysis of the sequence homology between ovine and bovine MHC regions demonstrated a remarkable conservation, with the overall homology reaching 86%. The actual level of homology could be higher because a number of gaps (over 10-40 kb) in the available bovine sequence contributed negatively to the homology score. For virtually any locus currently identified in bovine MHC, a homologous match could be identified in the ovine MHC, including those in the class IIb region (Figure 2). It is noteworthy that the ovine and bovine MHC class IIa and IIb regions exhibited exactly the same gene order and structural layout. In addition, the piece of non-MHC autonomic insertion between IIa and IIb was estimated to be of the same length (approximately 18.5 Mb) for both species. Furthermore, the order of bovine and ovine genetic loci within the inserted autonomic region was essentially the same as tested by over 120 SS-PCRs (data not shown). Taken together, these results support the hypothesis that cattle and sheep shared an ancestral chromosome containing the MHC before their divergence by evolution.

The hypothesis that cattle and sheep shared an ancestral chromosome was previously proposed in the studies of cattle [42-44]. Detailed mapping of BTA23 by radiation hybrid analysis [43,45] suggested that the ancestral MHC was likely disrupted by a large inversion that produced the bovine MHC class IIa and IIb regions. With the availability of detailed sequence information from the two ruminant species (bovine and ovine), the hypothesis has now gained additional support from the experimental data.

**Figure 3:**
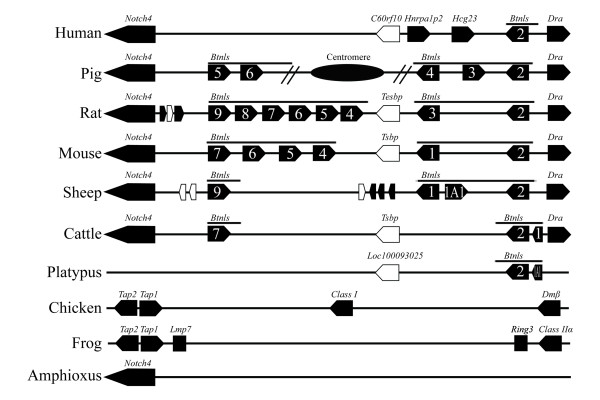
**A comparative alignment of *Btnl *loci among known MHC/loci of different species**. Solid and open box represent the known coding gene and predicted gene, respectively, with an arrow head of box indicating the orientation of gene transcription. A numeric number in a solid box indicates the gene family number of *Btnl *families. MHC of swine was interrupted by the Centromere as shown

Our sequence analysis also identified a *butyrophilin-like *(*Btnl *) cluster at the boundary between the ovine class IIa and III (Figure 3). *Banal *is critical for milk secretion and production [46]. Comparison of *Btnl *loci duplication showed that sheep has a moderate number of *Btnl *copies, more than that seen in platypus but less than those shown by mouse, rat or swine that have a larger litter sizes (Figure 3). *Btnl *is absent in non-mammal species like amphioxus, frog, and chicken, appears (*Btnl2*) in platypus, and is duplicated extensively in mammals that have more litter sizes. This might be an indication that milk production was closely associated with the function of MHC in mammals, due to the apparent need for mammals to protect their offspring from microbial infections via milk ingestion. Taken together, we propose a hypothesis that, formation of the *Btnl *loci is associated not only with the gene duplication of immunological loci, but also with the emergence of mammals in evolutionary history.

## Conclusion

A complete ovine MHC sequence map was assembled by successful shotgun sequencing of 26 overlapping BAC clones. This makes the sheep the second ruminant species for which the MHC sequence is available for evolutionary and functional studies. Gene annotation resulted in the identification of 177 genes, among which 145 were identified for the first time, and 10 were ovine-species specific. In addition, a total of 18 microRNAs coding sequences were predicted in the ovine MHC for the first time. Comparative analysis revealed a remarkable conservation of MHC sequence between sheep and cattle, supporting the hypothesis that the two species shared an ancestral chromosome that shaped the ruminant MHC as currently observed. Identification of a relatively large number of micro RNAs in the ovine MHC region helps to provide evidence that micro RNAs are actively involved in the regulation of MHC gene expression and function.

## Methods

### DNA shotgun sequencing

Shotgun sequencing libraries were constructed individually for each of the 26 BAC clones following the modified protocols described by Celera Genomics Group [47]. Briefly, *E. coli *stock containing the target BAC clones were used to prepare the BAC clone DNA, which were solicited to form randomized small DNA fragments between 0.5 - 2.0 kb. After cloning of the small fragments into the plasmids, random DNA sequencing was performed with an ABI 3730 automated DNA sequencers (Applied Biosystems, USA) to generate the randomized short DNA sequence reads.

### Assembling of BAC clone sequences

The short random DNA sequences generated by the sequencing were assembled into full-length sequence utilizing the Prep program (U.W., Seattle, WA, USA) for each of the BAC clones. Resequencing was performed when necessary for gaps detected during the sequence assembly, including sequencing by primer walking of the PCR-amplified fragments for regions showing low level of accuracy. Blast alignments [48] of the repeat-masked, assembled sequence against NCBI EST and non-redundant nucleotide databases were performed to identify expressed sequences and other highly conserved regions likely to contain functional genes.

### Sequence analysis

The assembled ovine MHC sequence was analyzed using an automatic Ensemble pipeline [49] with modifications to aid the manual duration process. Simple and interspersed repeats were detected using Tandem Repeats Finder [50] and Repeat Masker, respectively, using the mammalian library along with cow-specific repeats submitted to EMBL/NCBI/DDBJ. The combination of simple and interspersed repeats was used as a filter to mask the sequence during analysis. Novel genes or CDS loci were identified by having an open reading frame (ORF), plus certain similarity to the known genes or proteins. A predicted gene was defined by having high sequence homology to the predicted gene or ORF in other species. Pseudo genes were identified by sequence homology to known Pseudo genes (not shown). Comparative sequence alignments were performed using the waviest pipeline http://genome.lbl.gov/cgi-bin/WGVistaInput.

## Authors' contributions

KL carried out experiments, data analysis and gene annotation; JFG carried out conformational experiments; HBL and GL carried out construction of shotgun libraries; HTB assisted in manuscript writing, CFC and PPT performed data checking and technical assistance. RZM supervised the studies and wrote the manuscript. All authors read and approved the final manuscript.
